# The efficacy of mesenchymal stromal cell-derived therapies for acute respiratory distress syndrome—a meta-analysis of preclinical trials

**DOI:** 10.1186/s12931-020-01574-y

**Published:** 2020-11-20

**Authors:** Fengyun Wang, Bin Fang, Xinhua Qiang, Jingsong Shao, Lixin Zhou

**Affiliations:** Department of Critical Care Medicine, The First People’s Hospital of Foshan, Lingnan Avenue North 81, Shiwan, Chancheng, Foshan, 528000 China

**Keywords:** Acute lung injury, Acute respiratory distress syndrome, Conditioned medium, Extracellular vesicles, Exosomes, Microvesicles

## Abstract

**Background:**

The investigation of mesenchymal stromal cell (MSC)-conditioned medium or extracellular vesicles (exosomes or microvesicles) as a remedy for acute lung injury (ALI) or acute respiratory distress syndrome (ARDS) has become a fast-growing field in recent years. Our purpose was to conduct a meta-analysis to investigate the efficacy of MSC-derived therapies (MDTs) for ALI/ARDS in animal models.

**Methods:**

A meta-analysis of MDTs for ALI/ARDS in animal trials was performed. PubMed and EMBASE were searched to screen relevant preclinical trials with a predetermined search strategy.

**Results:**

A total of 17 studies that compared MDT with the ALI control group were included in our study. The pooled result derived from the comparison of the two groups suggested that MDT could significantly reduce the lung injury score (standardized mean difference (SMD) = − 4.02, 95% CI [− 5.28, − 2.23], P < 0.0001) and improve animal survival (OR = − 6.45, 95% CI [2.78, 14.97], P < 0.0001). MDT mitigated the infiltration of neutrophils in alveoli (SMD = − 3.38, 95% CI [− 4.58, − 2.18], P < 0.00001). MDT also reduced the wet-dry weight ratio of the lung (SMD = − 2.34, 95% CI [− 3.42, − 1.26], P < 0.0001) and the total protein in BALF (SMD = − 2.23, 95% CI [− 3.07, − 1.40], P < 0.00001). Furthermore, MDT was found to downregulate proinflammatory mediators such as IL-1, IL-6 and TNF-a and to upregulate anti-inflammatory mediators such as IL-10.

**Conclusion:**

MDT reduces lung injury and improves survival in animal ARDS models since it can ameliorate lung permeability, decrease inflammatory cell infiltration, downregulate proinflammatory mediators**,** and upregulate anti-inflammatory mediators. However, more animal studies and human trials are needed for further investigation.

## Introduction

In critically ill patients, ARDS is a severe clinical syndrome with high morbidity and mortality [1]. The pathophysiological features of ARDS are characterized by diffuse alveolar damage, acute noncardiogenic lung oedema, and decreased functional lung volume [2]. Patients with moderate and severe ARDS are usually in need of intubated mechanical ventilation. If exacerbated, the patients need be put in a prone position; alternatively, if the patient is unresponsive to regular treatment, ECMO should be employed as a salvage therapy [3]. While mechanical ventilation can provide urgently needed respiratory support, it can cause volutrauma, atelectrauma, and biotrauma, all of which may accentuate the condition of patients with ARDS [4]. To date, there are no evidence-based pharmaceutical agents for ARDS or any treatments directly targeting the pathophysiology of ARDS [5]. Mesenchymal stromal cells (MSCs)—a type of pluripotent stem cell—were first found in the bone marrow. With antibacterial, immunomodulatory, and tissue and organ repair and regeneration characteristics, MSCs have been widely investigated as a potential therapy in different scenarios for ALI/ARDS in the last few decades [6]. MSCs may be effective for ALI/ARDS caused by a variety of pathogenic factors, as they can ameliorate lung permeability, decrease inflammatory cell infiltration, downregulate inflammatory mediators and upregulate anti-inflammatory mediators. The effects of MSCs were assumed to be due to their engraftment and proliferation, which were demonstrated to be rather limited [7]; however, according to current research, the paracrine- and endocrine-related secretomes are more important for tissue damage repair [8–10]. MSCs may manage intracellular oxidative stress via exosomes, which can be engulfed and reutilized by macrophages, thus suppressing inflammation and regulating immunity; therefore, MSCs may have potential in lung injury treatment [11, 12]. However, MSCs may be oncogenic, triggering an immune response that, per se, may exacerbate ARDS in patients. Furthermore, the storage of MSCs may interfere with their gene expression or viability. As extracellular vesicles (EVs) are manufactured from conditioned medium (CM) by centrifugation, in this study, they are collectively referred to as MSC-derived therapies (MDTs). MDTs that contain these secretomes may have potential for treating ALI/ARDS. Today, MDT-related research is a fast-growing field [9, 13–15]. Although MDTs cannot proliferate like MSCs, they have the advantages of easier preservation and transfer. Furthermore, in comparison with MSCs, MDTs have reduced immunogenicity and are thus an attractive solution for allogeneic transplants [16]. We will investigate the efficacy of MDT for ALI/ARDS to evaluate whether it can improve survival, lower lung injury severity, and regulate immune balance via a meta-analysis of animal models.

## Methods

### Data sources

PubMed and EMBASE (up to February 14, 2020) were searched to screen relevant preclinical trials with an exquisitely crafted search strategy. Search terms included the following: acute respiratory distress syndrome, acute lung injury, mesenchymal stem cell, mesenchymal stromal cell, vesicles, microvesicles, exosome, and medium. The search strategy was as follows: ((((((((((((vesicles[Title/Abstract]) OR microvesicles[Title/Abstract]) OR ectosomes[Title/Abstract]) OR exosome[Title/Abstract]) OR nanoparticles[Title/Abstract]) OR microparticles[Title/Abstract]) OR exosomes[Title/Abstract]) OR oncosomes[Title/Abstract])) OR medium[Title/Abstract])) AND (((stem cell[Title/Abstract]) OR stromal cell[Title/Abstract]) OR msc[Title/Abstract])) AND ((((Acute Respiratory Distress Syndrome[Title/Abstract]) OR acute lung injury[Title/Abstract]) OR ARDS[Title/Abstract]) OR ALI[Title/Abstract]).

### Study selection

Two authors (FYW and BF) searched and screened the relevant literature independently and then checked the title and abstract of each retrieved article to decide which required further assessment. Full articles were retrieved if the titles and abstracts suggested that the study included a prospective design to investigate the therapeutic effects of MSC-derived therapy for ALI/ARDS in animal models. When there were disagreements, the two authors discussed them thoroughly to reach an agreement.

The inclusion criteria were as follows: (1) any controlled preclinical studies investigated MSC-derived therapy for ALI/ARDS; (2) any animal models of any species, age, or sex; and (3) MSC-derived therapy administered with any approach or any dosage. MSCs were defined using the minimal criteria set out in the International Society for Cellular Therapy (ISCT) consensus statement [17, 18].

The exclusion criteria were as follows: (1) non-interventional studies and (2) non-extractable data from a study that would prevent meta-analysis for at least one of the pre-specified outcomes.

### Qualitative assessment and data extraction

Two authors (WFY and FB) independently extracted data with a customized data extraction form and assessed the risk of bias. Study characteristics were extracted if they were related to the construct and external validity. The data extraction form included the following detailed information: (1) references and publication date; (2) species of animal; (3) lung injury model; (4) descriptions of the source of MSC; (5) the dose, and route of MSC derived therapy; and (6) the time points of assessment.

As the data in the literature were mostly presented as figures and not in numerical form, a validated open source graphical digitizer (WebPlot-Digitizer, version 4.2) was utilized to extract data from figures. First, the images of the figures for relevant outcomes from all included studies were saved as screenshots. Then, these images were uploaded into the programme to extract data. The first step was to define the type of graph analysed, usually a two-dimensional bar plot. Second, the axis was calibrated by assigning four points of known values. Then, the related data points were extracted and added by directly clicking on the graph; thereupon, WebPlot-Digitizer calculated the precise coordinates of each point, which in turn was used to calculate the mean and standard deviation for each variable.

### Data analysis and statistical methods

Data analyses of this review were performed by Review Manager 5.3. A heterogeneity assessment was performed via the χ^2^ test, where a p value less than 0.05 was considered significant. A funnel plot was applied to check for publication bias, and I^2^ was applied to estimate the total variation attributed to heterogeneity among studies. Values of I^2^ less than 25% were considered as having low heterogeneity, and a fixed-effect model for meta-analysis was used. Values of I^2^ no less than 25% represented moderate (25–75%) or high levels (> 75%) of heterogeneity existing among studies, and a random effects model was applied. For dichotomous variables, odds ratios (ORs) were used for statistical calculations, whereas for continuous variables, mean and standardized mean differences (SMDs) were used. All statistical tests were two-sided, and a p value of less than 0.05 was considered statistically significant.

### Primary and secondary outcomes

Our primary outcomes were lung injury score and survival. Secondary outcomes included inflammatory factors IL-1β, IL-6 and TNF-α, anti-inflammatory factor IL-10, lung wet weight to dry weight ratio (W/D ratio), total protein in BALF, and neutrophil counts in BALF.

## Results

### Study selection process

**Fig. 1:**
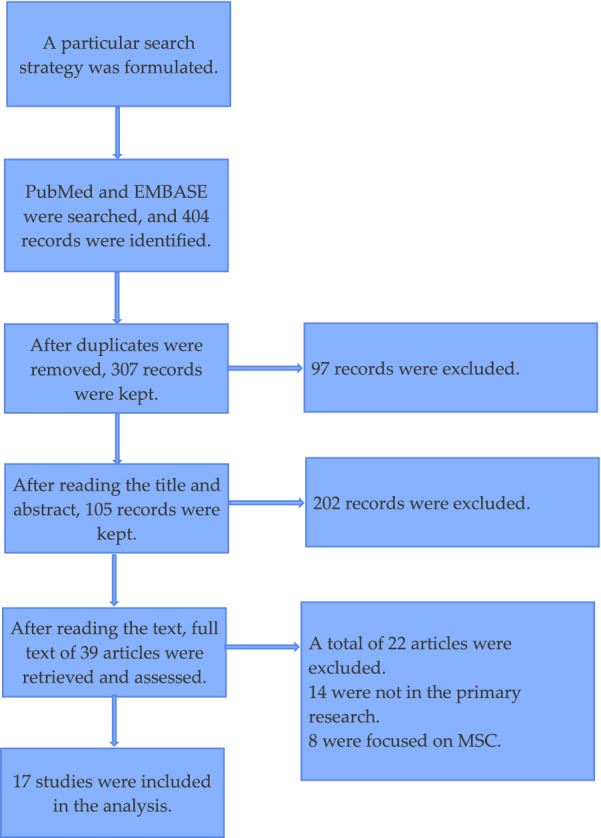
The flow diagram of the literature search process

Figure 1 is the flow diagram of the literature search process. In total, 404 articles were found by means of electronic database searches. After duplicates were deleted, a total of 307 articles were pooled to read the titles and abstracts**.** Of these, 202 articles were discarded, and 105 were retained for further evaluation. After reading the text of each paper, the full text of 39 articles was then retrieved for further assessment. Finally, 17 articles were found to have met the inclusion criteria [19–35].

### Characteristics of the included studies

All the included studies were conducted over the last few decades. The animal models were mostly established in mice and rats; only one study applied pigs. Intratracheal administration of LPS or *E. coli* to induce ALI was the most popular approach among the included studies. Bleomycin, VILI, H1N1 virus, and ischaemia–reperfusion were the respective causes of ALI in each study. The tissue sources of MSCs included bone marrow, umbilical cord, adipose tissue, and neural crest. The MDT doses were also diverse among the studies. Additionally, the outcome assessment time points of the studies differed significantly; most of them were completed within 3 days, while a few lasted up to 1 week. The detailed characteristics of the included studies are presented in Table 1.

**Table 1:** The characteristics of the included preclinical studies

Reference	Animal, gender	Injury model	MSCs source	Type of stem cell derived therapy	Time of assessment
Amir Varkouhi [19]	SD rats	*E. coli *(5. 109 CFUs)*, IT*	Human UC MSC	Extracellular vesicles, 100*10^6^/kg, IV	48 h after *E. coli* instillation
Antoine Monsel [20]	Male C57BL/6 mice	*E. coli* (2 or 3 × 10^6^ CFUs), IT	Human BM MSC	Microvesicles, 1*10^6^ cells/10 ul, IT/IV	18, 24, or 72 h after modeling
Chen Wenxia [21]	Male SD rats	BLM (4 mg/kg), IT	Human WJ MSC	Microvesicles, IT	48 h or 1 week after bleomycin treatment
Huang Ruoqiong [22]	C57BL/6 mice	LPS (4 mg/kg), IT	Human AD MSC	MSC extracellular vesicles, 100 μg/200 ul	48 h after LPS insult
James Devaney [23]	Male SD rats	*E. coli* (2 × 109), IT	Human BM MSC	hMSC-CM	48 h after E. coli instillation
Johnatas Silva [24]	C57BL/6 mice	LPS 2 mg/kg, IT	Mouse BM MSC	MSC extracellular vesicles, 1*10^5^ cells, IV	24 h after MSCs, or EV administration
Lavinia Ionescu [25]	Male C57BL/6 Mice	4 mg/kg LPS	Mouse BM MSC	MSC CM 250,000 cells/30 μl, IT	48 h post-LPS insult
Li Qing-Chun [26]	Male SD rats	Chest trauma induced ALI	Rat BM MSC	MSC-derived exosomes, 25 ug/100 μl, IV	7 days after modeling
Liu Jianpei [27]	Male SD rats	Intestinal IR induced ALI	Rat BM MSC	MSC-derived exosomes, 5–10 ug/500 μl, IV	20 h after modeling
Mahesh Khatri [28]	White-Duroc pigs	H1N1, 5 × 106 TCID50 per pig	Porcine BM MSC	MSC-derived extracellular vesicles, 10 μg/ml, IT	1 and 3 days post-infection
Mairead Hayes [29]	Male SD rats	Ventilator induced lung injury	Rat BM MSC	MSC-CM, 4 × 10^6^ cells/500 μl, IV	4 h following VILI induction
Peng Chung-Kan [30]	Male SD rats	IR induced ALI	RAT NC SCs	NCSCs-CM, 5 × 10^6^ cells/250 μl, IV	90 min after modeling
Tang Xiaodan [31]	Male C57BL/6 mice	LPS (4 mg/kg), IT	Human BM MSC	MSC microvesicles, 2 × 10^6^ cells/30 μl, IV	48 h after microvesicles injection
Vincent Su [32]	Male C57BL/6 mice	LPS (5 mg/kg), IT	Mouse MSC	MSC-CM, 200 μl, IV	24 h after MSC-CM treatment
Xu Ning [33]	Male SD rats	Phosgene (8.33 g/m3), inhaled	Rat BM MSC	MSC-derived exosomes, 50 mL, IT	6, 24, and 48 h post-exposure
Yi Xiaomeng [34]	C57BL/6 mice	LPS (1 mg/kg), IT	Mouse BM MSC	MSC-derived exosomes (30 μL), IV	24 h after LPS induction
Zhu Ying-gang [35]	Male C57BL/6 mice	LPS (4 mg/kg), IT	Human MSC	MSC microvesicles, 1.5 × 10^6^ cells/ 15 μl	48 h after microvesicles injection

*SD* Sprague–Dawley, *LPS* lipopolysaccharide, *CFU* colony forming unit, *IP* intra-peritoneal, *IT* intratracheal, *IV* intravenous, *BM* bone marrow, *UC* umbilical cord, *AD* adipose-derived, *MSC* mesenchymal stromal cells, *CM* conditoned medium

### Meta-analysis: MSC-derived therapies versus the ALI control group

#### Primary outcomes

##### Lung injury score and survival

**Fig. 2:**
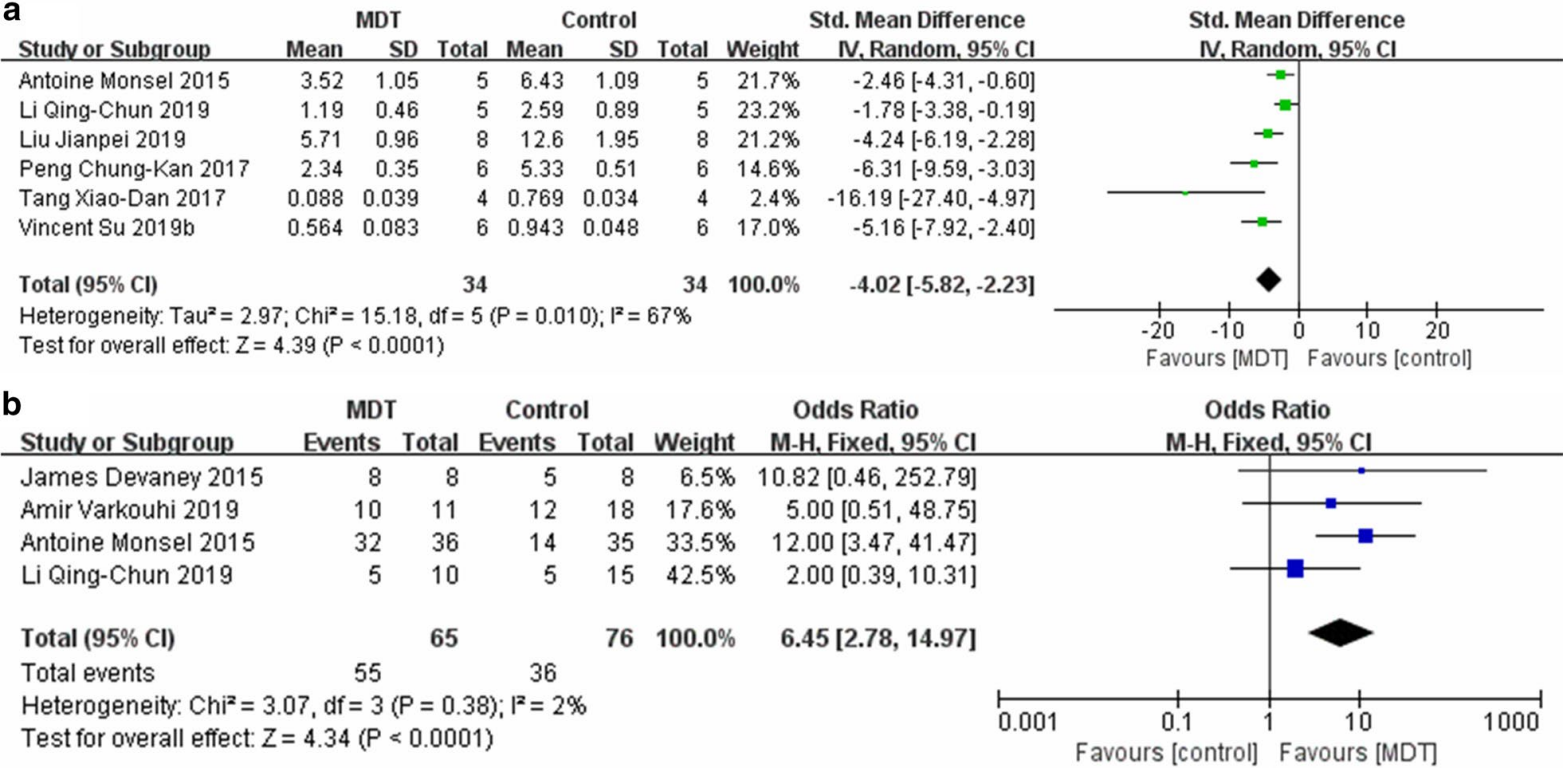
Main outcome of the meta-analyses of MDT compared with the ALI control group: **a** lung injury score; **b** survival. The size of each square represents the proportion of information given by each trial. Crossing with the vertical line suggests no difference between the two groups. *MDT* MSC-derived therapy, *ALI* acute lung injury, *IV* inverse variance, *CI* confidence interval, *M-H* Mantel-Haensze, *df* degree of freedom

A total of six studies investigated the lung injury score. The pooled result suggested that MDT, based upon comparison with the ALI control group, could significantly reduce the lung injury score, with a standardized mean difference (SMD) = − 4.02, 95% CI [− 5.28, − 2.23], P < 0.0001, and I^2^ = 67% (Fig. 2a). Four studies reported on post-injury survival. The synthesis of these results, derived from comparison with the ALI control group, indicated that MDT can significantly promote animal survival, with an OR = − 6.45, 95% CI [2.78, 14.97], P < 0.0001, and I^2^ = 2% (Fig. 2b).

#### Secondary outcomes

##### Neutrophil counting in BALF

**Fig. 3:**
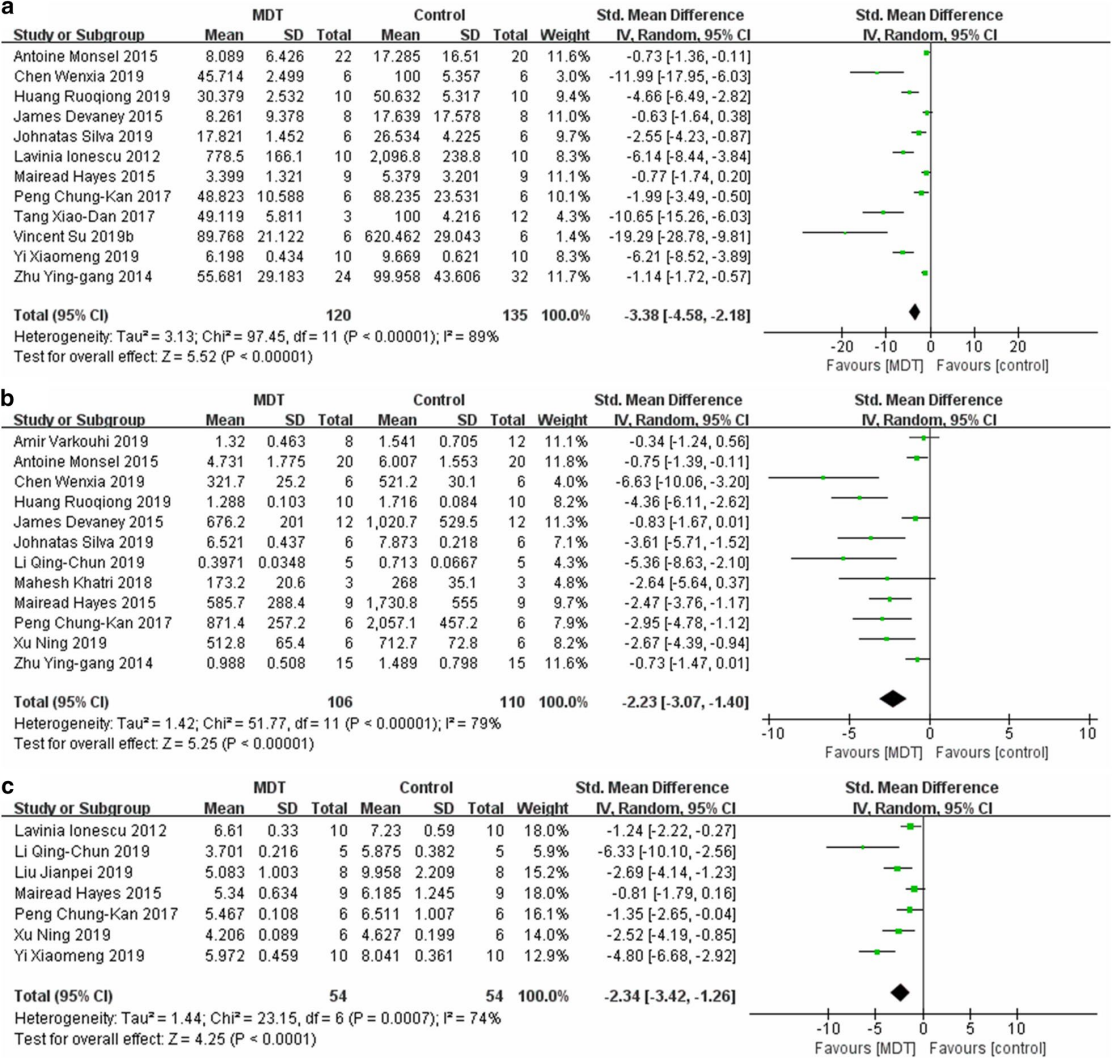
Meta-analyses of the neutrophils in BALF (**a**), total protein in BALF (**b**) and lung W/D ratio (**c**) between the MDT and ALI control groups. The size of each square represents the proportion of information given by each trial. Crossing with the vertical line suggests no difference between the two groups. *MDT* MSC-derived therapy, *ALI* acute lung injury, *IV* inverse variance, *CI* confidence interval, *M-H* Mantel-Haensze, *df* degree of freedom

Neutrophil counting in BALF was reported in a total of 11 studies. In comparison with the control group, MDT mitigated the infiltration of neutrophils in alveoli (SMD = − 3.38, 95% CI [− 4.58, − 2.18], P < 0.00001, I^2^ = 89%) (Fig. 3a).

##### Total protein level in BALF

In total, 12 studies investigated the total protein in BALF. Their pooled result indicated that, in comparison with the control group, MDT ameliorated protein leakage (SMD = − 2.23, 95% CI [− 3.07, − 1.40], P < 0.00001, I^2^ = 79%) (Fig. 3b).

##### Wet to dry weight ratio of lung (W/D ratio)

The synthesized results of 7 studies proved that MDT could reduce the W/D ratio when compared with the control group, SMD = -2.34, 95% CI [− 3.42, − 1.26], P < 0.0001, I^2^ = 74% (Fig. 3c).

##### Inflammatory and anti-inflammatory factors pertaining to lung injury.

**Fig. 4:**
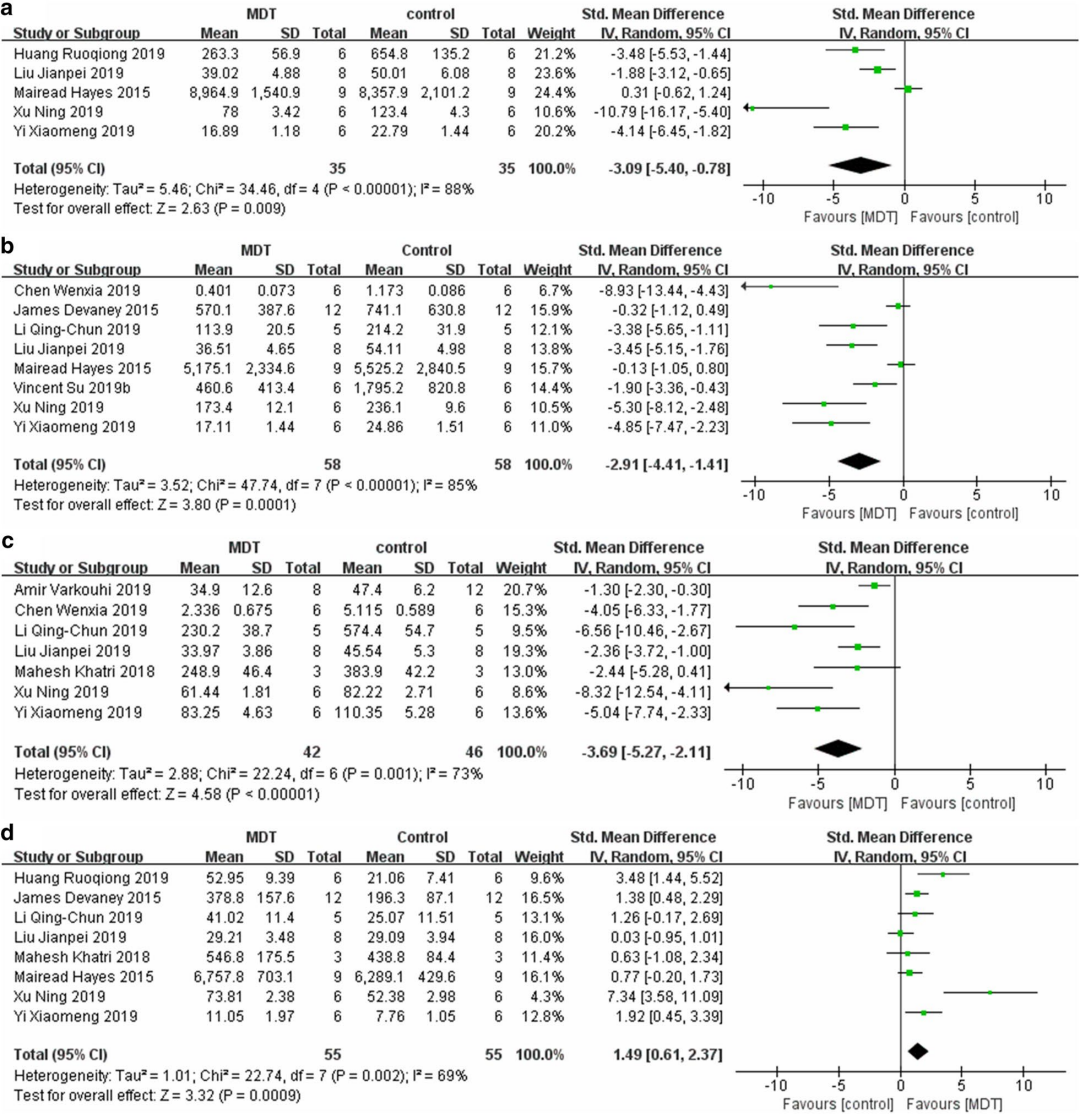
Meta-analyses of cytokines between the MDT and ALI control groups: **a** IL-1β, **b** IL-6, **c** TNF-α, and **d** IL-10. The size of each square represents the proportion of information given by each trial. Crossing with the vertical line suggests no difference between the two groups. *MDT* MSC-derived therapy, *ALI* acute lung injury, *IV* inverse variance, *CI* confidence interval, *M-H* Mantel-Haensze, *df* degree of freedom

A total of 5 studies investigated IL-1 in lung tissue. MDT was shown to decrease the level of IL-1 compared with that in the ALI control group (SMD = − 3.09, 95% CI [− 5.40, − 0.78], P = 0.0001, I^2^ = 88%) (Fig. 4a). A total of 8 studies reported IL-6, and their pooled result suggests that, in comparison with the control, MDT could reduce the level of IL-6 (SMD = − 2.91, 95% CI [− 4.41, − 1.41], P = 0.0001, I^2^ = 85%) (Fig. 4b). Additionally, 7 studies presented data about TNF-a, the synthetic result of which revealed that MDT could downregulate the level of TNF-a, with an SMD = − 3.69, 95% CI [− 5.27, − 2.11], P < 0.00001, and I^2^ = 73%, (Fig. 4c). The pooled results of 8 studies suggested that MDT upregulated the level of IL-10 (SMD = 1.49, 95% CI [0.61, 2.37], P = 0.0009, I^2^ = 69%) (Fig. 4d).

## Discussion

EVs comprising exosomes and microvesicles are extracted from conditioned medium (CM) through centrifugation; similar to CM, they also possess the MSC secretome [8, 36]. Thus, CM and EVs were integrated as MSC-derived therapies (MDTs) in this study, the purpose of which was to summarize the evidence of MDTs for ALI/ARDS. In our study, meta-analyses were conducted for parameters such as lung injury score (LIS), survival, neutrophils in BALF, protein in BALF, W/D ratio and inflammatory mediators. These thorough analyses were important and essential for demonstrating the efficacy of MDT for ALI/ARDS. To date, only a few similar studies regarding lung diseases have been conducted, and none of them have solely focused on ALI/ARDS [37, 38]. To our knowledge, this is the first meta-analysis focused on the efficacy of MDT for ALI/ARDS in preclinical studies.

Our meta-analysis demonstrated that MDT can mitigate the severity of ALI/ARDS in animal models. The lung injury score (LIS), a scoring scale under a microscope, is a widely used pathophysiological tool to assess lung injury severity in preclinical trials. In our study, the pooled result indicated that MDT significantly reduced LIS, which is direct evidence that MDT can attenuate lung injury severity. The results also suggested that, in animal trials, MDT was able to increase survival. Moreover, our study revealed that MDT can downregulate the levels of inflammatory factors such as IL-1, IL-6 and TFN-a while upregulating the level of IL-10, a well-known anti-inflammatory factor. Thus, MDT may regulate immunologic balance in a desirable manner. The immunomodulatory effects of MDT may be an important reason for ameliorated lung injury and improved survival.

The W/D ratio of the lung is an extensively utilized parameter to assess pulmonary vessel permeability in animal studies, which was demonstrated to be decreased in our study. This reduced ratio indicated that MDT can improve lung water clearance. Our meta-analysis suggests that MDT can downregulate the infiltration of neutrophils into the alveolar space. The decrease in neutrophils in alveoli not only attenuated inflammation and subsequent high vessel permeability in the lung but also reduced lung tissue damage, which in turn may improve the outcomes. In addition, our study discovered that the total protein in BALF was reduced with MDT treatment. The reduction in total protein was not just the consequence of downregulated lung vessel permeability but also may be the mechanism of improved lung compliance. As the “hyaline membrane” is a protein-rich fluid formed on the alveolar surface, it pathophysiologically increases alveolar interfacial tension and blocks oxygenation in ALI/ARDS.

EVs can be divided into exosomes, microvesicles, or apoptotic bodies depending on size, biogenesis, and composition. Exosomes are generally homogenous in size, with a diameter ranging from 40 to 200 nm, whereas microvesicles are relatively heterogeneous, ranging from 50 to 1000 nm in diameter up to the state of the cell during release [8, 39]. Furthermore, the process of vesicle formation and release from cells also differs between exosomes and microvesicles. Each of the EV subtypes has its own characteristic surface and intracellular markers. Although exosomes, microvesicles and conditioned medium may externally differ from one another, in our study, the available subgroup analyses of each subtype demonstrated that they were consistently efficacious for ALI/ARDS in preclinical trials. The probable reason for this consistency is that they all internally contain the therapeutic secretome which is irrelevant to size or formation. Specifically, exosome (EX), microvesicle (MV) and conditioned medium (CM) subgroup meta-analyses were available for outcomes such as LIS, neutrophil counting in BALF, total protein in BALF, IL-6 in the lung and IL-10 in the lung. Only the IL-6 subgroup meta-analysis detected statistically significant difference between the EX and CM subgroups. Whether this difference was generated by confounding factors or by original efficacious differences remains to be further studied. The detailed subgroup meta-analysis results can be found in the Additional file 1.

ARDS is a common clinical syndrome that causes respiratory distress due to refractory hypoxia for a variety of heterogeneous aetiologies. The hallmark of ARDS is noncardiogenic lung oedema, a result of diffuse alveolar damage, increased permeability of lung vessels, infiltration of inflammatory cells, and protein-rich fluid leakage into the alveolar space, which causes overwhelming hypoxia [2]. The popularity of lung protective ventilation [40, 41], mainly characterized by low tidal volume and low inspiratory pressure, decreased ARDS mortality in the early 2000s [42, 43]. Furthermore, in 2013, an RCT discovered that prone positioning can significantly reduce 28-d and 90-d mortality on the basis of lung protective ventilation [44]. The control of driving pressure was also associated with increased survival in ventilator settings in ARDS [45]. Other measures taken for respiratory support, such as lung recruitment and PEEP titration, may increase mortality; thus, they are not recommended in the clinical routine [46]. Although increased understanding of ARDS has been achieved in recent decades, no pharmaceutical agents have been verified as effective treatments. Trials for medications such as aspirin, intravenous salbutamol, recombinant human keratinocyte growth factor, rosuvastatin, and simvastatin were all ineffective because they did not result in reduced mortality of ARDS [5].

To date, with regard to treating ARDS, there is no targeted medicine that has proven to be effective [47]. Since 2007, a large body of preclinical trials have investigated the efficacy of MSC therapy for ALI/ARDS, demonstrating that MSCs can stabilize the alveolar-capillary barrier, enhance alveolar fluid clearance, and decrease infection and inflammation [48–50]. Microvesicles derived from stem cells were reported to contain secretomes, such as protein and mRNA components that are crucial for stem cell renewal and expansion [51]. Since MSCs have been revealed to have the potential to treat ALI/ARDS, conditioned medium (CM) or extracellular vesicles (EVs) of MSCs, which possess these secretomes, have been the subjects of studies in recent years.

In basic numerical research, MSCs exhibit lung protective potential via paracrine growth and anti-inflammatory factors and downregulation of inflammatory pathways. Not only were MSC intensively investigated in vivo and in vitro in preclinical trials, several human trials, regarding the safety of MSC’s for ARDS, were also carried out in the past few years [52–54]. Although the safety of MSCs has been questioned because of their oncogenic possibility, to date, no direct MSC-related adverse events have been detected in the above trials. The safety of MDT should be more reassuring since no live cells were transplanted during the treatment [55]. According to Katie Famous et al., clinically, ARDS can be divided into two subphenotypes, which have different inflammation statuses and respond differently to fluid infusion [56]. Whether these distinct ARDS subphenotypes respond differently to MSCs or MDTs is a topic worthy of future research.

There are several limitations in our meta-analysis. First, the overall sample size of our study was small due to the small sample size of preclinical trials. Second, the causes of ALI/ARDS were not unanimous within the studies. Third, the sources of MDT were not consistent within the studies; therefore, those of both human and animal origin were investigated. Additionally, the dosage of MDT and the intervention duration also varied among the studies. These limitations may generate substantial heterogeneity among the studies, which may thereby confound the results of our analyses. Finally, the lack of large animal trials and regular clinical parameters (such as respiratory mechanics) in the included MDT trials may miss some important information useful to guide its application in clinical settings.

## Conclusion

MDT reduced lung injury and improved survival in animal ALI/ARDS models via the following mechanisms: ameliorating lung permeability, decreasing inflammatory cell infiltration, downregulating proinflammatory mediators**,** and upregulating anti-inflammatory mediators. However, more large-animal studies and human trials are needed for further investigation.

## Supplementary information

**Additional file 1.** Subgroup meta-analysis.

## Data Availability

The datasets used and/or analysed in this study are available from the corresponding author upon reasonable request.

## Abbreviations

ARDS: Acute respiratory distress syndrome
ALI: Acute lung injury
LPS: Lipopolysaccharide
BM: Bone marrow
UC: Umbilical cord
AD: Adipose-derived
MSCs: Mesenchymal stem cells

## References

[1] Bellani G, Laffey JG, Pham T, Fan E, Brochard L, Esteban A, Gattinoni L, van Haren F, Larsson A, McAuley DF (2016). Epidemiology, patterns of care, and mortality for patients with acute respiratory distress syndrome in intensive care units in 50 countries. JAMA.

[2] Huppert LA, Matthay MA, Ware LB (2019). Pathogenesis of acute respiratory distress syndrome. Semin Respir Crit Care Med.

[3] Matthay MA, Zemans RL, Zimmerman GA, Arabi YM, Beitler JR, Mercat A, Herridge M, Randolph AG, Calfee CS (2019). Acute respiratory distress syndrome. Nat Rev Dis Primers.

[4] Curley GF, Laffey JG, Zhang H, Slutsky AS (2016). Biotrauma and ventilator-induced lung injury: clinical implications. Chest.

[5] Fan E, Brodie D, Slutsky AS (2018). Acute respiratory distress syndrome: advances in diagnosis and treatment. JAMA.

[6] Lee JW, Fang X, Gupta N, Serikov V, Matthay MA (2009). Allogeneic human mesenchymal stem cells for treatment of *E. coli* endotoxin-induced acute lung injury in the ex vivo perfused human lung. Proc Natl Acad Sci U S A.

[7] Togel F, Hu Z, Weiss K, Isaac J, Lange C, Westenfelder C (2005). Administered mesenchymal stem cells protect against ischemic acute renal failure through differentiation-independent mechanisms. Am J Physiol Renal Physiol.

[8] Lee JH, Park J, Lee JW (2019). Therapeutic use of mesenchymal stem cell-derived extracellular vesicles in acute lung injury. Transfusion.

[9] Gunawardena TNA, Rahman MT, Abdullah BJJ, Abu Kasim NH (2019). Conditioned media derived from mesenchymal stem cell cultures: the next generation for regenerative medicine. J Tissue Eng Regen Med.

[10] van Balkom BWM, Gremmels H, Giebel B, Lim SK (2019). Proteomic signature of mesenchymal stromal cell-derived small extracellular vesicles. Proteomics.

[11] Phinney DG, Di Giuseppe M, Njah J, Sala E, Shiva S, St Croix CM, Stolz DB, Watkins SC, Di YP, Leikauf GD (2015). Mesenchymal stem cells use extracellular vesicles to outsource mitophagy and shuttle microRNAs. Nat Commun.

[12] Najar M, Raicevic G, Fayyad-Kazan H, Bron D, Toungouz M, Lagneaux L (2016). Mesenchymal stromal cells and immunomodulation: a gathering of regulatory immune cells. Cytotherapy.

[13] Monsel A, Zhu YG, Gudapati V, Lim H, Lee JW (2016). Mesenchymal stem cell derived secretome and extracellular vesicles for acute lung injury and other inflammatory lung diseases. Expert Opin Biol Therapy.

[14] Wakayama H, Hashimoto N, Matsushita Y, Matsubara K, Yamamoto N, Hasegawa Y, Ueda M, Yamamoto A (2015). Factors secreted from dental pulp stem cells show multifaceted benefits for treating acute lung injury in mice. Cytotherapy.

[15] Wuchter P, Bieback K, Schrezenmeier H, Bornhauser M, Muller LP, Bonig H, Wagner W, Meisel R, Pavel P, Tonn T (2015). Standardization of Good Manufacturing Practice-compliant production of bone marrow-derived human mesenchymal stromal cells for immunotherapeutic applications. Cytotherapy.

[16] Mardpour S, Hamidieh AA, Taleahmad S, Sharifzad F, Taghikhani A, Baharvand H (2019). Interaction between mesenchymal stromal cell-derived extracellular vesicles and immune cells by distinct protein content. J Cell Physiol.

[17] Dominici M, Le Blanc K, Mueller I, Slaper-Cortenbach I, Marini F, Krause D, Deans R, Keating A, Prockop D, Horwitz E (2006). Minimal criteria for defining multipotent mesenchymal stromal cells. The International Society for Cellular Therapy position statement. Cytotherapy.

[18] Galipeau J, Krampera M, Barrett J, Dazzi F, Deans RJ, DeBruijn J, Dominici M, Fibbe WE, Gee AP, Gimble JM (2016). International Society for Cellular Therapy perspective on immune functional assays for mesenchymal stromal cells as potency release criterion for advanced phase clinical trials. Cytotherapy.

[19] Varkouhi AK, Jerkic M, Ormesher L, Gagnon S, Goyal S, Rabani R, Masterson C, Spring C, Chen PZ, Gu FX (2019). Extracellular vesicles from interferon-gamma-primed human umbilical cord mesenchymal stromal cells reduce escherichia coli-induced acute lung injury in rats. Anesthesiology.

[20] Monsel A, Zhu YG, Gennai S, Hao Q, Hu S, Rouby JJ, Rosenzwajg M, Matthay MA, Lee JW (2015). Therapeutic effects of human mesenchymal stem cell-derived microvesicles in severe pneumonia in mice. Am J Respir Crit Care Med.

[21] Chen W, Wang S, Xiang H, Liu J, Zhang Y, Zhou S, Du T, Shan L (2019). Microvesicles derived from human Wharton's Jelly mesenchymal stem cells ameliorate acute lung injury partly mediated by hepatocyte growth factor. Int J Biochem Cell Biol.

[22] Huang R, Qin C, Wang J, Hu Y, Zheng G, Qiu G, Ge M, Tao H, Shu Q, Xu J (2019). Differential effects of extracellular vesicles from aging and young mesenchymal stem cells in acute lung injury. Aging (Albany NY).

[23] Devaney J, Horie S, Masterson C, Elliman S, Barry F, O'Brien T, Curley GF, O'Toole D, Laffey JG (2015). Human mesenchymal stromal cells decrease the severity of acute lung injury induced by *E. coli* in the rat. Thorax.

[24] Silva JD, de Castro LL, Braga CL, Oliveira GP, Trivelin SA, Barbosa-Junior CM, Morales MM, Dos Santos CC, Weiss DJ, Lopes-Pacheco M (2019). Mesenchymal stromal cells are more effective than their extracellular vesicles at reducing lung injury regardless of acute respiratory distress syndrome etiology. Stem Cells Int.

[25] Ionescu L, Byrne RN, van Haaften T, Vadivel A, Alphonse RS, Rey-Parra GJ, Weissmann G, Hall A, Eaton F, Thebaud B (2012). Stem cell conditioned medium improves acute lung injury in mice: in vivo evidence for stem cell paracrine action. Am J Physiol Lung Cell Mol Physiol.

[26] Li QC, Liang Y, Su ZB (2019). Prophylactic treatment with MSC-derived exosomes attenuates traumatic acute lung injury in rats. Am J Physiol Lung Cell Mol Physiol.

[27] Liu J, Chen T, Lei P, Tang X, Huang P (2019). Exosomes released by bone marrow mesenchymal stem cells attenuate lung injury induced by intestinal ischemia reperfusion via the TLR4/NF-kappaB pathway. Int J Med Sci.

[28] Khatri M, Richardson LA, Meulia T (2018). Mesenchymal stem cell-derived extracellular vesicles attenuate influenza virus-induced acute lung injury in a pig model. Stem Cell Res Therapy.

[29] Hayes M, Curley GF, Masterson C, Devaney J, O’Toole D, Laffey JG (2015). Mesenchymal stromal cells are more effective than the MSC secretome in diminishing injury and enhancing recovery following ventilator-induced lung injury. Intensive Care Med Exp.

[30] Tang XD, Shi L, Monsel A, Li XY, Zhu HL, Zhu YG (2017). Mesenchymal stem cell microvesicles attenuate acute lung injury in mice partly mediated by Ang-1 mRNA. Stem Cells.

[31] Peng CK, Wu SY, Tang SE, Li MH, Lin SS, Chu SJ, Huang KL (2017). Protective effects of neural crest-derived stem cell-conditioned media against ischemia-reperfusion-induced lung injury in rats. Inflammation.

[32] Su VY, Lin CS, Hung SC, Yang KY (2019). Mesenchymal stem cell-conditioned medium induces neutrophil apoptosis associated with inhibition of the NF-kappaB pathway in endotoxin-induced acute lung injury. Int J Mol Sci.

[33] Xu N, Shao Y, Ye K, Qu Y, Memet O, He D, Shen J (2019). Mesenchymal stem cell-derived exosomes attenuate phosgene-induced acute lung injury in rats. Inhal Toxicol.

[34] Yi X, Wei X, Lv H, An Y, Li L, Lu P, Yang Y, Zhang Q, Yi H, Chen G (2019). Exosomes derived from microRNA-30b-3p-overexpressing mesenchymal stem cells protect against lipopolysaccharide-induced acute lung injury by inhibiting SAA3. Exp Cell Res.

[35] Zhu YG, Feng XM, Abbott J, Fang XH, Hao Q, Monsel A, Qu JM, Matthay MA (2014). Human mesenchymal stem cell microvesicles for treatment of *Escherichia coli* endotoxin-induced acute lung injury in mice. Stem Cells.

[36] Shah TG, Predescu D, Predescu S (2019). Mesenchymal stem cells-derived extracellular vesicles in acute respiratory distress syndrome: a review of current literature and potential future treatment options. Clin Transl Med.

[37] Emukah C, Dittmar E, Naqvi R, Martinez J, Corral A, Moreira A, Moreira A (2019). Mesenchymal stromal cell conditioned media for lung disease: a systematic review and meta-analysis of preclinical studies. Respir Res.

[38] Mohammadipoor A, Antebi B, Batchinsky AI, Cancio LC (2018). Therapeutic potential of products derived from mesenchymal stem/stromal cells in pulmonary disease. Respir Res.

[39] Huppert LA, Liu KD, Matthay MA (2019). Therapeutic potential of mesenchymal stromal cells in the treatment of ARDS. Transfusion.

[40] Amato MB, Barbas CS, Medeiros DM, Magaldi RB, Schettino GP, Lorenzi-Filho G, Kairalla RA, Deheinzelin D, Munoz C, Oliveira R (1998). Effect of a protective-ventilation strategy on mortality in the acute respiratory distress syndrome. N Engl J Med.

[41] Acute Respiratory Distress Syndrome N, Brower RG, Matthay MA, Morris A, Schoenfeld D, Thompson BT, Wheeler A. Ventilation with lower tidal volumes as compared with traditional tidal volumes for acute lung injury and the acute respiratory distress syndrome. N Engl J Med. 2000; 342:1301–1308. 10.1056/NEJM200005043421801 10793162

[42] Brower RG, Lanken PN, MacIntyre N, Matthay MA, Morris A, Ancukiewicz M, Schoenfeld D, Thompson BT, National Heart L, Blood Institute ACTN (2004). Higher versus lower positive end-expiratory pressures in patients with the acute respiratory distress syndrome. N Engl J Med.

[43] Fan E, Del Sorbo L, Goligher EC, Hodgson CL, Munshi L, Walkey AJ, Adhikari NKJ, Amato MBP, Branson R, Brower RG (2017). An official American Thoracic Society/European Society of Intensive Care Medicine/Society of Critical Care Medicine Clinical Practice Guideline: mechanical ventilation in adult patients with acute respiratory distress syndrome. Am J Respir Crit Care Med.

[44] Guerin C, Reignier J, Richard JC, Beuret P, Gacouin A, Boulain T, Mercier E, Badet M, Mercat A, Baudin O (2013). Prone positioning in severe acute respiratory distress syndrome. N Engl J Med.

[45] Amato MB, Meade MO, Slutsky AS, Brochard L, Costa EL, Schoenfeld DA, Stewart TE, Briel M, Talmor D, Mercat A (2015). Driving pressure and survival in the acute respiratory distress syndrome. N Engl J Med.

[46] Cavalcanti AB, Suzumura EA, Laranjeira LN, Paisani DM, Damiani LP, Guimaraes HP, Romano ER, Regenga MM, Taniguchi LNT, Writing Group for the Alveolar Recruitment for Acute Respiratory Distress Syndrome Trial I (2017). Effect of lung recruitment and titrated positive end-expiratory pressure (PEEP) vs low PEEP on mortality in patients with acute respiratory distress syndrome: a randomized clinical trial. JAMA.

[47] Lewis SR, Pritchard MW, Thomas CM, Smith AF (2019). Pharmacological agents for adults with acute respiratory distress syndrome. Cochrane Database Syst Rev.

[48] Mei SH, McCarter SD, Deng Y, Parker CH, Liles WC, Stewart DJ (2007). Prevention of LPS-induced acute lung injury in mice by mesenchymal stem cells overexpressing angiopoietin 1. PLoS Med.

[49] Gupta N, Su X, Popov B, Jae WL, Serikov V, Matthay MA (2007). Intrapulmonary delivery of bone marrow-derived mesenchymal stem cells improves survival and attenuates endotoxin-induced acute lung injury in mice. J Immunol.

[50] Matthay MA, Thompson BT, Read EJ, McKenna DH, Liu KD, Calfee CS, Lee JW (2010). Therapeutic potential of mesenchymal stem cells for severe acute lung injury. Chest.

[51] Ratajczak J, Miekus K, Kucia M, Zhang J, Reca R, Dvorak P, Ratajczak MZ (2006). Embryonic stem cell-derived microvesicles reprogram hematopoietic progenitors: evidence for horizontal transfer of mRNA and protein delivery. Leukemia.

[52] Zheng G, Huang L, Tong H, Shu Q, Hu Y, Ge M, Deng K, Zhang L, Zou B, Cheng B, Xu J (2014). Treatment of acute respiratory distress syndrome with allogeneic adipose-derived mesenchymal stem cells: a randomized, placebo-controlled pilot study. Respir Res.

[53] Wilson JG, Liu K, Zhuo H, Caballero L, McMillan M, Fang X, Cosgrove K, Vojnik R, Calfee CS, Lee JW (2015). Mesenchymal stem (stromal) cells for treatment of ARDS: a phase 1 clinical trial. Am J Respir Crit Care Med.

[54] Matthay MA, Calfee CS, Zhuo H, Thompson BT, Wilson JG, Levitt JE, Rogers AJ, Gotts JE, Wiener-Kronish JP, Bajwa EK (2019). Treatment with allogeneic mesenchymal stromal cells for moderate to severe acute respiratory distress syndrome (START study): a randomised phase 2a safety trial. Lancet Respir Med.

[55] Rani S, Ryan AE, Griffin MD, Ritter T (2015). Mesenchymal stem cell-derived extracellular vesicles: toward cell-free therapeutic applications. Mol Ther.

[56] Famous KR, Delucchi K, Ware LB, Kangelaris KN, Liu KD, Thompson BT, Calfee CS, Network A (2017). Acute respiratory distress syndrome subphenotypes respond differently to randomized fluid management strategy. Am J Respir Crit Care Med.

