# Supplementary material for: The efficacy of mesenchymal stromal cell-derived therapies for acute respiratory distress syndrome—a meta-analysis of preclinical trials
Source: Respir Res. 2020 Nov 20;21:307. doi: 10.1186/s12931-020-01574-y (PMC7677103; doi:10.1186/s12931-020-01574-y)
Supplement: Supplementary file 1 — Additional file 1. Subgroup meta-analysis. [file 12931_2020_1574_MOESM1_ESM.docx]

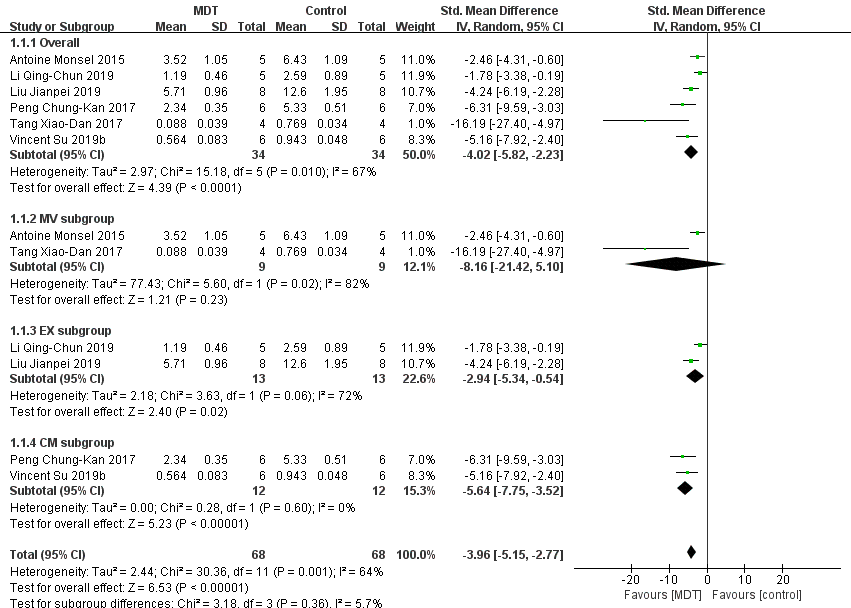


1. The subgroup meta-analysis of lung injury score that compares MSC’s derived therapy (MDT) with acute lung injury (ALI) control group. The analysis didn’t detect any statistically significant difference among the microvesicles(MV), exosomes(EX), and conditioned medium (CM) subgroups (p=0.36).


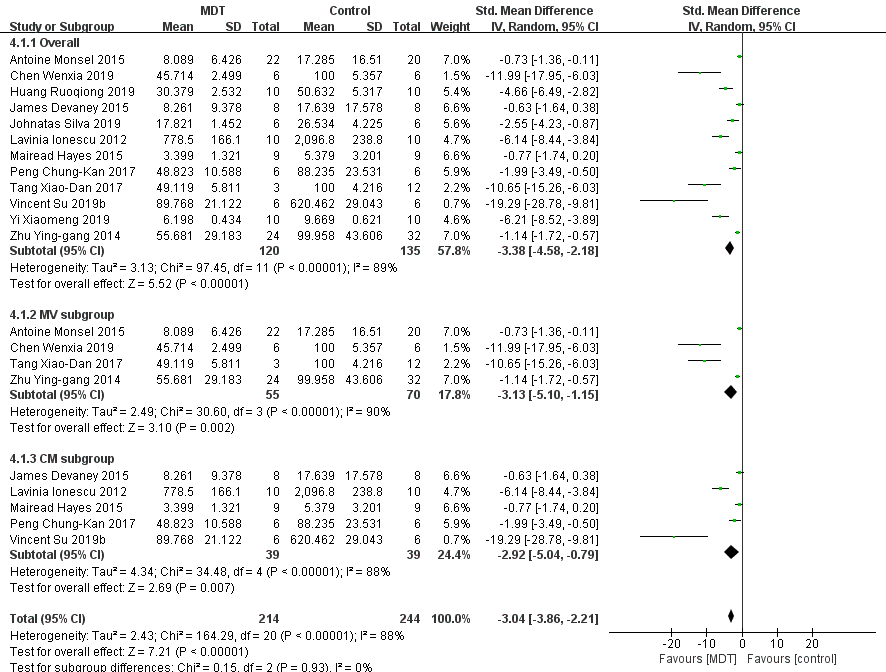


1. The subgroup meta-analysis of neutrophil counting in BALF that compares the MDT with the ALI control group. The analysis didn’t detect any statistically significant difference between the MV and the CM subgroups (p=0.93).


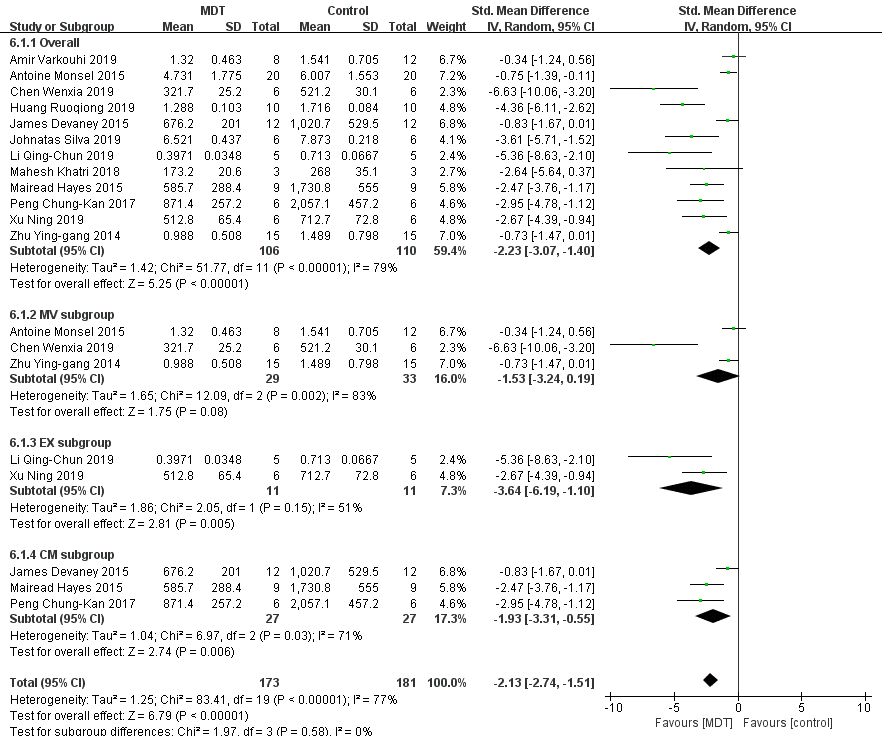


1. The subgroup meta-analysis of total protein in BALF that compares the MDT with the ALI control group. The analysis didn’t detect any statistically significant difference among the MV, the EX and the CM subgroups (p=0.58).


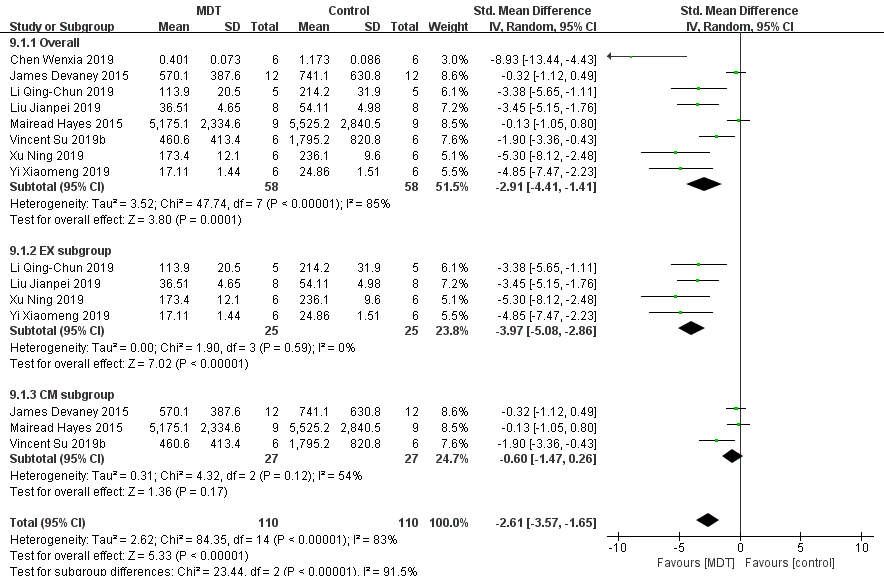


1. The subgroup meta-analysis of IL-6 level that compares the MDT with the ALI control group. The analysis detected statistically significant difference between the EX and the CM subgroups (p<0.00001).


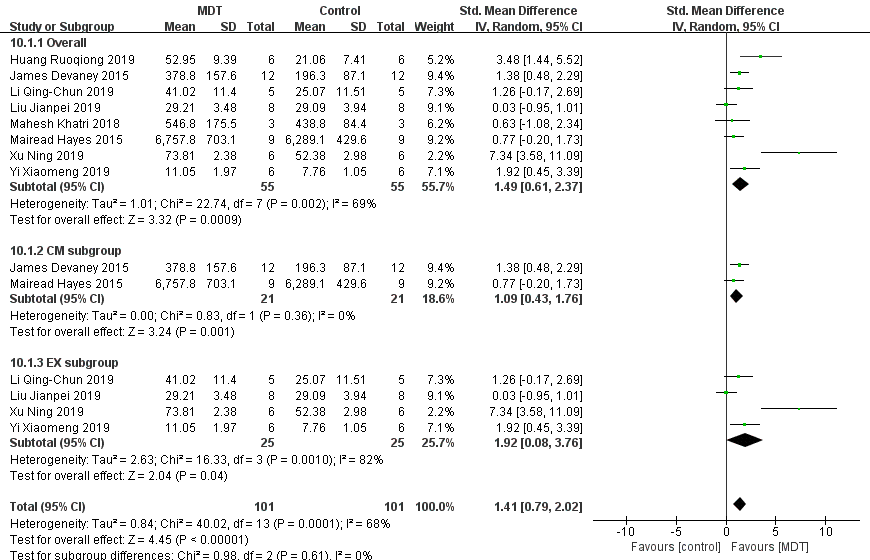


1. The subgroup meta-analysis of IL-10 that compares the MDT with the ALI control group. The analysis didn’t detect any statistically significant difference between the EX and the CM subgroups (p=0.61).
